# Developing an Intervention Framework to Prevent Surgical Complications in Patients With Metabolic Syndrome: A Qualitative Study of Health Professional Perspectives on Barriers and Enablers

**DOI:** 10.1111/ans.70786

**Published:** 2026-06-08

**Authors:** Philip Norris, Jeff Gow, Thomas Arthur, Daevyd Rodda, Joseph Coory, Florin Oprescu, Stephen Neville, Nicholas Ralph

**Affiliations:** ^1^ School of Nursing and Midwifery University of Southern Queensland Toowoomba Australia; ^2^ School of Commerce University of Southern Queensland Toowoomba Australia; ^3^ School of Accounting, Economics and Finance University of KwaZulu‐Natal Durban South Africa; ^4^ Colorectal and General Surgeon, St Andrew's Toowoomba Hospital Toowoomba Australia; ^5^ Orthopaedic Surgeon, Sunshine Coast Orthopaedic Group, Sunshine Coast University Private Hospital Birtinya Australia; ^6^ School of Health University of the Sunshine Coast Sippy Downs Australia

**Keywords:** adverse events, behaviorally informed framework, intervention development, metabolic syndrome, operative risk factors, perioperative care, postoperative complications, qualitative study, surgical outcomes, theoretical domains framework

## Abstract

**Background:**

Metabolic syndrome (MetS) is a cluster of risk factors, including cardiovascular disease, diabetes, chronic hypertension, obesity, and hypercholesterolemia, which collectively increase the risk of chronic diseases and adverse health outcomes. Affecting up to 30% of the global population, MetS poses significant challenges for surgical patients, with emerging evidence indicating poorer postoperative outcomes compared to nonaffected populations. Despite its growing prevalence, there is a lack of dedicated interventions to address the specific needs of this group. This study aims to explore the barriers and facilitators influencing the management of MetS in surgical patients, with the goal of informing intervention development.

**Methods:**

A qualitative, exploratory study was conducted using the Theoretical Domains Framework (TDF) to guide data collection and analysis. Semi‐structured interviews were conducted with clinicians involved in the perioperative care of patients with MetS, focusing on their perspectives on barriers and facilitators to effective management. Data were analyzed thematically and mapped to the 14 TDF domains.

**Results:**

Clinicians identified significant barriers, including resource constraints, fragmented care pathways, and variability in team roles. Facilitators included peer validation, multidisciplinary collaboration, and the integration of decision‐support tools. Emotional and motivational factors also emerged as critical determinants of engagement.

**Conclusion:**

This study underscores the need for targeted interventions to address the unique challenges of managing MetS in surgical contexts. The findings provide a foundation for developing behaviorally informed frameworks, with implications for improving surgical outcomes and reducing healthcare costs.

## 1 Introduction

Metabolic syndrome (MetS) represents a constellation of interconnected metabolic abnormalities, including central obesity, dyslipidaemia, hypertension, and impaired glucose metabolism, that collectively heighten the risk of cardiovascular disease and diabetes [1]. Globally, up to 30% of adults are affected by MetS, with prevalence steadily increasing due to rising rates of obesity and sedentary lifestyles [2]. Within surgical populations, MetS poses unique challenges, significantly increasing the risk of postoperative complications such as infections, impaired wound healing, venous thromboembolism, and extended recovery periods [3]. Patients with MetS have been found to experience higher postoperative morbidity compared to individuals without the syndrome, with estimates varying across procedures and populations [4]. These risks are compounded by the syndrome's complex pathophysiology, which disrupts multiple physiological systems and requires a multidisciplinary approach to management [5].

The mechanisms underlying these adverse outcomes are multifactorial. Obesity, a hallmark of MetS, contributes to chronic low‐grade inflammation, which amplifies pro‐inflammatory cytokine activity and compromises tissue repair [6]. Insulin resistance and hyperglycaemia further exacerbate cellular dysfunction, impairing energy utilization and wound healing [7]. Dyslipidaemia, another critical component of MetS, is closely linked to endothelial dysfunction, which increases susceptibility to thromboembolic complications [8]. Additionally, hypertension places undue stress on cardiovascular and renal systems, reducing physiological reserves and elevating perioperative risks [9]. As the global burden of MetS grows, addressing these multifaceted challenges becomes increasingly critical, particularly in surgical contexts where even minor complications can have cascading effects on recovery and long‐term health. Evidence from a recent systematic review of 13 million individual surgical patients highlights that patients with MetS undergoing surgery face a significantly higher risk of postoperative complications, including infections, delayed wound healing, and extended recovery periods, underscoring the urgent need for interventions targeting this high‐risk group [10].

Despite its well‐documented impact on surgical outcomes, interventions to mitigate the risks associated with MetS are underdeveloped. Traditional perioperative care often focuses on surgical technique and acute management, neglecting the behavioral and contextual factors that influence patient outcomes. Effective interventions require a comprehensive understanding of these factors, particularly those that shape adherence to preoperative and postoperative management practices [11]. This study aligns with the first stage of the Medical Research Council (MRC) framework for developing complex interventions, which emphasizes the importance of identifying key behavioral determinants through theory‐driven, exploratory research [12]. Specifically, the use of qualitative methods provides a means to investigate clinicians' perspectives, offering nuanced insights into the barriers and facilitators of effective management of MetS in surgical contexts [13].

The Theoretical Domains Framework (TDF) offers a robust foundation for understanding these behavioral determinants. Developed as an integrative model, the TDF consolidates constructs from 33 behavioral theories into 14 domains, providing a systematic approach to exploring factors that influence behavior [14]. These domains include Knowledge, Skills, Social/Professional Role and Identity, Beliefs About Capabilities, Beliefs About Consequences, Motivation and Goals, Memory, Attention, and Decision Processes, Environmental Context and Resources, Social Influences, Emotion, Behavioral Regulation, Optimism, Reinforcement, and Intentions [14, 15]. Each domain captures a unique aspect of behavior change, enabling researchers to identify specific barriers and facilitators that can be targeted through interventions. For example, the Knowledge domain addresses clinicians' understanding of MetS‐related risk, while the Environmental Context and Resources domain examines external factors such as access to services and organizational support [16].

This study applies the TDF to explore the behavioral and contextual factors influencing the management of MetS in surgical patients. Qualitative data, collected from healthcare professionals involved in perioperative care, were coded thematically and mapped to the TDF domains to identify actionable insights for intervention development. This approach not only provides a comprehensive understanding of the factors influencing practice but also facilitates the design of evidence‐based strategies tailored to the perioperative management of MetS [17]. Notably, this research addresses a critical gap in the literature by focusing on behavioral determinants within the perioperative context, an area often overlooked in traditional biomedical frameworks [18].

The objectives of this study are threefold. First, it seeks to identify the barriers and facilitators of effective MetS management in the perioperative period, mapped to the TDF domains. Second, it draws on clinicians' reflections on a proposed intervention framework to inform its refinement for perioperative practice. Finally, the study provides evidence‐informed recommendations for designing and implementing interventions that improve surgical outcomes for patients with MetS. By addressing these objectives, this research contributes to the growing body of evidence on the importance of behavioral science in surgical care and supports the development of more effective, implementation‐ready approaches.

## 2 Methods

This exploratory qualitative study was conducted to explore the barriers and facilitators influencing the management of metabolic syndrome (MetS) in surgical patients, with the aim of informing intervention development. The study design was guided by the first stage of the Medical Research Council (MRC) framework for developing and evaluating complex interventions, which emphasizes the importance of identifying key behavioral determinants and understanding context through exploratory research [12]. The Theoretical Domains Framework (TDF) was employed as a conceptual tool to systematically examine the behavioral and contextual factors impacting perioperative care for patients with MetS [14].

### 2.1 Study Design and Setting

An exploratory qualitative research design was selected for its ability to provide rich, contextualized insights into the experiences and perspectives of healthcare professionals. Consistent with a qualitative descriptive approach, as articulated by Sandelowski, this design was chosen to provide a comprehensive, practice‐oriented account of clinicians' perspectives without imposing high levels of theoretical interpretation [19]. Semi‐structured interviews were conducted to allow for flexibility in exploring individual viewpoints while ensuring alignment with the TDF domains. Interviews were conducted within perioperative services in Australian hospital settings where clinicians routinely manage surgical patients with metabolic syndrome, providing access to a diverse group of clinicians with direct experience managing MetS in surgical contexts.

### 2.2 Participants

Participants included healthcare professionals directly involved in the perioperative care of surgical patients with MetS. Participants were identified through invitations distributed via Australian surgical, nursing, and allied health professional colleges, requesting that members nominate clinicians with expertise in, or a demonstrated track record of, managing metabolic syndrome in surgical patients. Respondents who expressed interest were contacted by the research team and an interview was scheduled at a time of their convenience. Purposive sampling was used to ensure variation in professional roles and expertise, capturing a broad spectrum of experiences relevant to the research objectives [20]. Participants included surgeons [*n* = 12], anesthetists [*n* = 2], Registered Nurses/Nurse Practitioners [*n* = 3], and one dietitian, physiotherapist, and occupational therapist respectively. All participants held a minimum of 5 years' clinical experience and considered themselves to hold expertise in the delivery of preoperative, intraoperative, or postoperative care for patients with MetS. Inclusion criteria focused on professionals with substantial involvement in the design or delivery of perioperative care pathways relevant to MetS risk management. A total of 20 participants were recruited. Recruitment continued until no substantively new themes were identified during concurrent data collection and analysis, consistent with accepted approaches to assessing thematic saturation [21].

### 2.3 Data Collection

Data were collected through one‐on‐one, in‐depth interviews conducted in private settings to encourage open discussion and ensure confidentiality. Interviews were conducted by PN and NR, both experienced qualitative researchers. Neither interviewer had a preexisting professional or personal relationship with any participant. Participants were also presented with a preliminary version of the intervention framework, and their feedback was incorporated to refine its components. The interviews were guided by a structured protocol informed by the 14 domains of the TDF. Questions were designed to elicit participants' perspectives on the knowledge, skills, roles, and contextual factors influencing MetS management. Example questions included:

- “What do you think are the advantages of managing risks in patients with metabolic syndrome? Are there any disadvantages?”
- “How confident are you in your ability to implement a framework for managing risks in patients with metabolic syndrome? What aspects are you least confident about?”
- “What resources would you need to implement this framework in your practice? Are these resources currently available?” [15]

All interviews were audio‐recorded with participants' consent and transcribed verbatim for analysis.

### 2.4 Data Analysis

Data analysis followed a hybrid deductive‐inductive approach. The 14 domains of the Theoretical Domains Framework served as a deductive organizing structure [15], with thematic analysis conducted within each domain following Braun and Clarke's six‐step procedural framework [22] and drawing on grounded theory coding principles for the move from codes to themes [23]. Following familiarization with the transcripts, data were assigned to one or more TDF domains. Within each domain, inductive open coding was used to identify the dimensions, factors, and conditions shaping participants' accounts. These were then synthesized into a core theme intended to encapsulate the unifying analytic account of what participants conveyed in relation to that domain, with the contributing dimensions retained and elaborated in the narrative results. Where coders identified candidate elements that could plausibly stand as parallel themes, an analytic decision was made as to whether these constituted distinct themes or dimensions of a core theme; this judgment was made on the basis of whether a unifying analytic story was present. Transcripts were coded independently by PN and NR. The two coders met to compare coding decisions and discuss theme development, including disagreements about whether elements should be treated as separate themes or as dimensions within a core theme. Discrepancies were resolved through discussion until consensus was reached; a third coder was available for adjudication but was not required. Data relating to feedback on the preliminary intervention framework were analyzed alongside interview data and used to iteratively refine the framework in alignment with the identified behavioral and contextual determinants.

### 2.5 Ethical Considerations

The study received ethical approval from the institutional ethics committee, and all participants provided written informed consent (University of Southern Queensland Human Research Ethics Committee Approval Number ETH2023‐0005). Confidentiality was maintained by anonymizing transcripts and securely storing data in accordance with data protection guidelines. The study adhered to established principles of ethical research practice, including respect for participant autonomy and use of data solely for research purposes.

## 3 Results

The results are deductively mapped to the 14 domains of the Theoretical Domains Framework (TDF), providing a structured synthesis of the barriers and facilitators identified by clinicians managing patients with metabolic syndrome (MetS) in surgical contexts. These findings reflect clinicians' professional perspectives, including their observations of patient behaviors as perceived by clinicians, team dynamics, and systemic challenges in the perioperative setting.

### 3.1 Knowledge

Clinicians demonstrated a high level of awareness regarding the critical role of MetS management in improving surgical outcomes. Participants consistently highlighted that addressing modifiable risk factors, such as obesity, hypertension, and glucose dysregulation, leads to fewer complications, shorter hospital stays, and reduced healthcare costs. However, gaps were noted in disseminating evidence‐based knowledge across multidisciplinary teams. Clinicians expressed concern that variations in training and education limited their ability to adopt standardized protocols, particularly for complex cases. They emphasized the need for accessible, streamlined frameworks to enhance knowledge translation within and across teams.

### 3.2 Skills

A diverse set of clinical skills was identified as essential for managing patients with MetS effectively. Participants emphasized the importance of proficiency in risk stratification, prehabilitation planning, and intraoperative risk management. While most clinicians felt confident in performing these tasks, some noted significant challenges in communication and interdisciplinary collaboration around treating patients with MetS. These confidence issues were most pronounced around referring surgeons to allied health professionals such as dietitians and physiotherapists. Advanced training in nutritional and pharmacological management was highlighted as a priority for addressing complex patient needs. Clinicians suggested that formalized skill development programs and team‐based workshops could improve overall competency and confidence.

### 3.3 Beliefs About Capabilities

Clinicians' confidence in implementing MetS management protocols varied depending on the stage of care. Participants expressed high confidence in screening patients and managing intraoperative risks, where established guidelines and workflows were available. This confidence was expressed across professional groups, including surgeons and anesthetists with intraoperative responsibilities, as well as nursing and allied health participants involved in preoperative assessment, screening, and planning. However, they reported lower confidence in coordinating prehabilitation and postoperative care, citing barriers such as resource limitations, fragmented care pathways, and time constraints. Participants suggested that clear decision‐support tools and defined care protocols could significantly enhance their perceived capabilities in addressing these gaps.

### 3.4 Beliefs About Consequences

Participants strongly agreed on the adverse outcomes associated with suboptimal management of MetS. Observed consequences included increased morbidity, prolonged recovery times, and greater economic burdens on healthcare systems. Clinicians also identified the consequences of not treating MetS symptoms preoperatively, often resulting in avoidable surgical delays and readmissions. These observations reinforced the need for integrated care pathways that address modifiable risk factors to improve both patient outcomes and operational efficiency.

### 3.5 Motivation and Goals

Motivation to implement improved MetS management frameworks was driven by both intrinsic and extrinsic factors. Intrinsic motivation stemmed from clinicians' desire to optimize patient outcomes, reduce complications, and enhance professional satisfaction. Extrinsic motivators included achieving institutional objectives, such as improving care quality metrics and reducing hospital costs. However, competing priorities, such as managing high patient volumes, sometimes tempered enthusiasm for adopting new frameworks. Clinicians noted that tangible evidence of success—such as reduced complication rates or positive patient feedback—would help sustain their motivation and commitment.

### 3.6 Environmental Context and Resources

The systemic and environmental factors influencing MetS management were among the most frequently discussed barriers. Clinicians consistently highlighted the need for dedicated infrastructure, such as prehabilitation centers and integrated electronic health records, to streamline workflows and improve care delivery. Financial constraints were identified as a major obstacle, with limited funding available for pilot programs, staff training, and resource allocation. Participants emphasized that organizational support and policy alignment were critical for addressing these challenges, particularly in demonstrating the long‐term cost‐efficiency of improved MetS management strategies.

### 3.7 Professional Role and Identity

Clinicians identified the surgical team as central to the implementation of MetS management frameworks. Surgeons were perceived as key leaders, responsible for driving change and coordinating care. However, participants noted variability in the roles and responsibilities of other team members, such as anesthetists and allied health professionals. This lack of clarity sometimes hindered effective collaboration. Clinicians advocated for well‐defined roles and responsibilities within multidisciplinary teams to foster accountability and streamline communication.

### 3.8 Social Influence

Social influences played a significant role in shaping clinicians' willingness to adopt new MetS management frameworks. Participants emphasized the importance of peer validation and shared learning, noting that successful implementation in other institutions often inspired confidence in their own efforts. Collaborative networks and inter‐institutional partnerships around improving MetS care around surgery were identified as valuable resources for sharing best practices and fostering a culture of collective improvement.

### 3.9 Memory, Attention, and Decision Processes

Clinicians highlighted the need for systematic prompts and structured decision‐making pathways to ensure consistent identification and management of MetS. The lack of standardized processes often led to variability in care delivery, with some clinicians relying on informal cues rather than evidence‐based guidelines. Participants suggested that electronic health record alerts, visual aids, and standardized protocols could significantly improve adherence to best practices and reduce cognitive load in high‐pressure environments.

### 3.10 Emotion

Emotional factors were frequently identified as barriers to effective MetS management. Clinicians described feelings of frustration and stress stemming from the complexity of managing MetS within resource‐constrained settings. Participants also expressed empathy for patients, recognizing that the demands of prehabilitation and lifestyle changes could be overwhelming. These emotional dimensions underscored the need for supportive strategies, including counseling and motivational interventions, to address both clinician burnout and patient resistance.

### 3.11 Behavioral Regulation

Participants noted that tools for self‐monitoring, such as weight‐tracking apps and patient progress logs, were valuable facilitators of adherence. These tools allowed clinicians to identify trends, monitor patient progress, and intervene early when necessary. However, inconsistent use of these tools, their application to MetS and surgery, and the lack of standardized protocols often limited their effectiveness. Clinicians suggested that embedding self‐monitoring strategies into routine surgical workflows could enhance their utility and impact.

### 3.12 Optimism

Clinicians expressed varying levels of optimism regarding the potential benefits of implementing structured MetS management frameworks. Those with prior experience in similar initiatives were generally more confident in the feasibility and outcomes of such efforts. Conversely, clinicians working in resource‐constrained environments were more skeptical, emphasizing the importance of demonstrating tangible benefits, such as improved patient outcomes or reduced costs, to build optimism and engagement.

### 3.13 Reinforcement

The role of reinforcement in facilitating behavior change was underexplored in the interviews. However, participants suggested that incorporating positive reinforcement strategies, such as recognition programs, verbal praise, and tangible rewards, could help sustain motivation among staff. They also noted that patient adherence might be improved through incentivized participation in prehabilitation programs.

### 3.14 Intentions

Clinicians consistently expressed strong intentions to adopt improved frameworks for MetS management. However, they acknowledged that these intentions were often difficult to translate into sustained behavior change due to systemic barriers, such as time pressures and limited organizational support. Participants emphasized the need for structured implementation plans, ongoing training, and regular performance evaluations to bridge the gap between intention and action.

**FIGURE 1:**
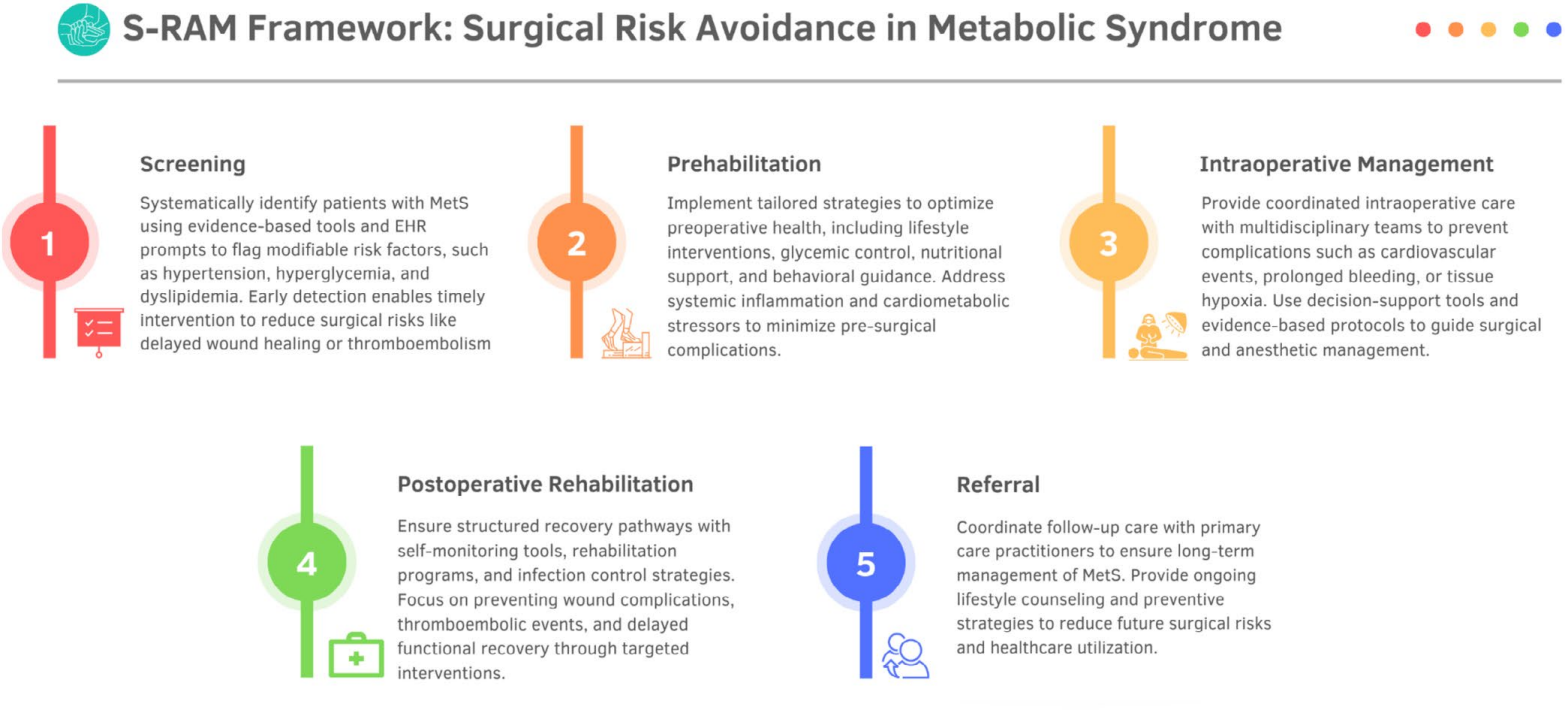
Surgical risk avoidance in metabolic syndrome (S‐RAM) framework.

The qualitative findings, mapped to the domains of the Theoretical Domains Framework (TDF), highlighted key barriers and facilitators that shaped the development of the intervention framework. Table 1 summarizes these themes with representative quotes. Based on these insights and end‐user feedback, the framework was refined (see Figure 1) to integrate behavioral, systemic, and procedural strategies aimed at addressing surgical risks associated with MetS and improving patient outcomes.

**TABLE 1:** Theoretical domains framework representative themes and quotes.

TDF domain	Theme	Representative quote
Knowledge	Awareness of risks	*Well, the evidence is its better surgical outcomes, you know, lower mortality rates, better patient outcomes in general, patients will have less complication and infections after surgeries*.
Skills	Communication & interdisciplinary collaboration	*Good communication skills, particularly with patients to avoid weight stigmatization and to help them understand the benefits of delaying an operation*.
Beliefs about capabilities	Confidence in intraoperative management	*Confident in screening and intraoperative management*.
Beliefs about consequences	Increased risk of complications	*Increased morbidity, increased length of stay in hospital, increased return to theater, increased surgical site infections*.
Motivation and goals	Intrinsic motivation to improve outcomes	*Optimizing them from a metabolic syndrome perspective not only benefits them for this operation, it benefits them lifelong*.
Environmental context and resources	Need for improved technological resources	*The tech component as a resource is necessary having technology to do the screening that links with electronic medical records*.
Professional role and identity	Surgical team as central coordinators	*The surgical team would need to be on board and would need to be perceived as the drivers and leaders*.
Social influence	Increased likelihood due to peer adoption	*Yeah, if it was being successfully utilized elsewhere, it does make the implementation a lot easier*.
Memory, attention, and decision processes	Need for systematic prompts and pathways	*If you had a prompt or something that automatically, you know, in the electronic health record that prompted people to consider metabolic syndrome*.
Emotion	Overwhelm as a barrier	*It's just too much to manage all at once; we need better ways to prioritize*.
Behavioral regulation	Clinicians' perspectives on patient self‐monitoring as a facilitator of adherence	*When patients use tracking tools consistently, it's easier to identify issues early*.
Optimism	Confidence from prior successes	*I've seen this work before, and I think we can make it work here too*.
Reinforcement	Role of rewards in motivation	*Small incentives can make a big difference in getting patients to stay on track*.
Intentions	Intentions not translating to actions	*I want to implement this, but the daily demands make it hard to focus on long‐term changes*.

## 4 Discussion

This qualitative study provides critical insights into the experiences and perceptions of clinicians to inform the development and refinement of interventions for managing MetS in surgical patients. By applying the Theoretical Domains Framework (TDF), the study uses a theory‐informed approach to systematically identify barriers and facilitators to effective care, providing a basis for targeting these determinants in subsequent intervention design and refinement. The inclusion of clinician feedback during the iterative refinement of the intervention framework supported alignment with real‐world practices, which may enhance feasibility for implementation [24]. Unlike research focused solely on clinical outcomes, this study emphasizes behavioral and systemic determinants that shape perioperative practice. Engaging clinicians as stakeholders and end‐users may strengthen the relevance and acceptability of the proposed framework, reflecting an iterative refinement process grounded in clinical expertise [25]. Findings highlight the absence of dedicated perioperative frameworks for addressing MetS, despite its established association with increased postoperative morbidity, prolonged hospital stays, and higher healthcare costs [3]. Moreover, findings from this study align with a recent systematic review highlighting the need for targeted interventions for surgical patients with MetS [10]. That review demonstrated consistently higher complication rates in this population, reinforcing the importance of addressing behavioral and systemic determinants through structured care frameworks. By centring on clinicians' insights, this study informs the design of interventions that address systemic challenges and behavioral considerations relevant to surgical care.

Clinicians emphasized the importance of addressing MetS as a modifiable risk factor, reflecting its role as a contributor to surgical complications [3]. While awareness of MetS‐related risks was high, disparities in knowledge dissemination and training highlighted ongoing barriers to consistent application of evidence‐based practices. These findings align with previous research identifying gaps in guideline implementation and the need for coordinated education strategies to support multidisciplinary teams [21]. Similarly, while confidence in screening and intraoperative management was strong, prehabilitation and postoperative care were identified as areas of uncertainty, constrained by resource limitations and fragmented care pathways [15].

Social and environmental factors played a prominent role in shaping clinicians' engagement with MetS management practices. Peer validation and observed success in other institutions emerged as important motivators, consistent with literature describing the influence of social norms in healthcare settings [14]. Conversely, limited organizational support, lack of dedicated infrastructure, and poor integration of technological solutions were perceived as barriers to implementation. Prior research has demonstrated that electronic health record (EHR) prompts and decision‐support tools can improve adherence to best practices by reducing cognitive burden and standardizing workflows [26]. Clinicians in this study similarly highlighted the potential value of such tools in facilitating consistent and efficient perioperative care.

Motivational and emotional factors further influenced clinicians' engagement with MetS management frameworks. Intrinsic motivation, driven by a desire to improve patient outcomes, was often tempered by practical constraints, including high patient volumes and competing clinical demands. Emotional challenges, such as frustration and stress, were commonly described, particularly in contexts where systemic limitations constrained clinicians' capacity to optimize care. These findings are consistent with broader literature on clinician burnout, which links resource constraints and workload pressures to reduced engagement and well‐being [27]. Addressing these factors through supportive strategies, including recognition and feedback on impact, may help sustain engagement over time [17].

The approach adopted in this study aligns with the MRC framework for developing complex interventions, which emphasizes theory‐informed and stakeholder‐engaged design in early‐phase research [12]. Building on these findings, subsequent stages should focus on piloting and evaluating a multidisciplinary intervention framework that incorporates the workflows, decision‐support mechanisms, and behavioral strategies identified as salient in this study. Such work may draw on evidence from other chronic disease contexts, including diabetes and cardiovascular disease, where multidisciplinary approaches have been associated with improved outcomes and system efficiency [28].

Co‐design with broader stakeholder groups, including health service managers and allied health professionals, will be important for ensuring feasibility and sustainability at scale. Collaborative development processes may support alignment with organizational priorities and resource constraints [25]. Consideration of staffing, prehabilitation infrastructure, and technological integration will be essential to address the structural barriers identified by participants.

Behavioral science principles, including goal setting, feedback, and self‐monitoring, represent potential mechanisms to address cognitive and emotional barriers identified in this study. Tools such as weight‐tracking applications and patient progress logs, discussed by participants, have shown promise in improving engagement in other settings [29]. Evaluation of their integration within perioperative pathways will be necessary to determine acceptability, effectiveness, and unintended consequences.

Several areas warrant further investigation. TDF domains that were less prominent in this dataset, including reinforcement and optimism, may benefit from focused exploration in future studies. Longitudinal research will be required to assess the impact of MetS management frameworks on clinical outcomes, service efficiency, and patient‐reported outcomes. Incorporating patient and caregiver perspectives in future work will also be important to complement clinician‐identified determinants and support comprehensive intervention design.

This study contributes to the literature by using a theory‐informed qualitative approach to identify behavioral and contextual determinants relevant to perioperative management of MetS. By grounding framework development in clinicians' perspectives, it provides an empirically informed foundation for subsequent intervention testing. The findings underscore the importance of multidisciplinary collaboration, organizational support, and technological integration in addressing MetS‐related surgical risk and inform next steps toward implementation and evaluation.

## 5 Conclusion

Metabolic syndrome (MetS) poses significant challenges in surgical contexts, contributing to increased morbidity, prolonged recovery, and higher healthcare costs, yet there remains a lack of structured perioperative approaches specifically addressing MetS‐related risk. This study provides an early, theory‐informed contribution by identifying key behavioral, contextual, and systemic determinants influencing the management of MetS in surgical patients, as perceived by clinicians. The findings underscore the importance of multidisciplinary collaboration, with clearly defined roles, streamlined workflows, and targeted training to support consistent risk management across the perioperative continuum. Participants highlighted the potential value of integrating technological supports, such as decision‐support tools and electronic health record prompts, alongside behavioral strategies that address motivational and emotional barriers to practice change. Consistent with the MRC framework for complex intervention development, the next phase of work should focus on refining and pilot‐testing the proposed framework in real‐world settings, with iterative evaluation and stakeholder engagement to inform future feasibility and effectiveness studies. By grounding intervention development in clinician‐identified determinants, this research provides a foundation for subsequent implementation and evaluation of behaviorally informed approaches to improving perioperative care for patients with MetS.

## Author Contributions

**Thomas Arthur:** writing – review and editing, writing – original draft. **Daevyd Rodda:** writing – original draft. **Nicholas Ralph:** conceptualization, investigation, funding acquisition, writing – original draft, writing – review and editing, project administration, formal analysis, data curation, supervision, visualization, validation, methodology, resources. **Philip Norris:** conceptualization, investigation, writing – original draft, methodology, validation, visualization, writing – review and editing, formal analysis, project administration. **Jeff Gow:** conceptualization, methodology, writing – review and editing, supervision. **Stephen Neville:** writing – review and editing, writing – original draft, validation, methodology, supervision. **Joseph Coory:** writing – review and editing, writing – original draft. **Florin Oprescu:** writing – review and editing, writing – original draft, supervision.

## Funding

The authors have nothing to report.

## Data Availability

The data that support the findings of this study are available on request from the corresponding author. The data are not publicly available due to privacy or ethical restrictions.
